# Clot Meniscus Sign Is Associated With Thrombus Permeability and Choice of Mechanical Thrombectomy Technique in Acute Middle Cerebral Artery Occlusion

**DOI:** 10.3389/fneur.2022.850429

**Published:** 2022-02-24

**Authors:** Chuang Nie, Zhiming Kang, Mengqi Tu, Xiangbo Wu, Dong Sun, Bin Mei

**Affiliations:** ^1^Department of Neurology, Zhongnan Hospital of Wuhan University, Wuhan University, Wuhan, China; ^2^Department of Radiology, Zhongnan Hospital of Wuhan University, Wuhan University, Wuhan, China

**Keywords:** meniscus sign, thrombus permeability, mechanical thrombectomy, contact aspiration, stent retriever

## Abstract

**Background and Purpose:**

The method of mechanical thrombectomy (MT) is related to vascular anatomy and stroke etiology. Meniscus sign and thrombus permeability as imaging markers may be instructive for the selection of MT. This study aims to clarify the relationship among meniscus sign, thrombus permeability, and choice of MT in patients with acute middle cerebral artery occlusion.

**Materials and Methods:**

A total of 111 patients with acute middle cerebral artery occlusion (MCAO) who underwent MT were retrospectively analyzed. Clot meniscus sign was defined as the appearance of meniscoid/edge-like or single- or double-wall contrast channels besides or around insular blood clots. The radiographic, clinical, and surgical data of patients with MCAO with or without meniscus sign were compared.

**Results:**

The meniscus sign positive group (*n* = 26) has higher thrombus permeability (HUs) (26.92 ± 9.69 vs. 22.84 ± 7.88, *p* = 0.031) than those without it. Shorter puncture-to-recanalization (P2R) time (65.5 vs. 88, *p* = 0.012), higher complete recanalization rate (85.71 vs. 33.33%, *p* < 0.01), and better clinical outcome (*p* < 0.01) were obtained by selecting contact aspiration (CA) over stent retriever (SR) in patients with positive meniscus sign. In patients with negative meniscus sign, there was no significant difference in clinical outcome after receiving CA or SR.

**Conclusion:**

Patients with MCAO with positive meniscus sign have higher thrombus permeability and are more suitable for CA to acquire better clinical outcomes.

## Introduction

Since 2015, a series of clinical trials has been demonstrating great advantages of mechanical thrombectomy (MT) in treatment of patients with acute large vessel occlusion (1). In 2018, with publication of the results of the DAWN and DEFUSE-3 trials, the time window for endovascular treatment was extended from 6 to 24 h (2, 3). Complete recanalization directly affects the clinical benefit and prognosis of patients who underwent MT (4).

Stent retriever (SR) and contact aspiration (CA) are techniques now widely used in MT. However, differences in vascular anatomy of anterior and posterior circulation and classification of stroke etiology may lead to different results when choosing SR or CA. Thrombus permeability, as an index of pre-procedural imaging, is considered to be associated with etiological classification of stroke and the rate of first-pass recanalization in patients who underwent CA (5, 6). As a special occlusive clot sign in intraprocedural imaging, meniscus sign is considered to be related to the mechanism of embolism (7, 8). Compared with the regular occlusion subtype, the meniscus sign usually indicates higher recanalization rate, fewer operations, and better clinical outcomes (7, 9).

In this study, we aimed to compare the thrombus permeability, recanalization efficacy, and clinical outcome of SR vs. CA in patients with acute middle cerebral artery occlusion suggested by the presence of meniscus sign.

## Materials and Methods

### Study Design

**Figure 1:**
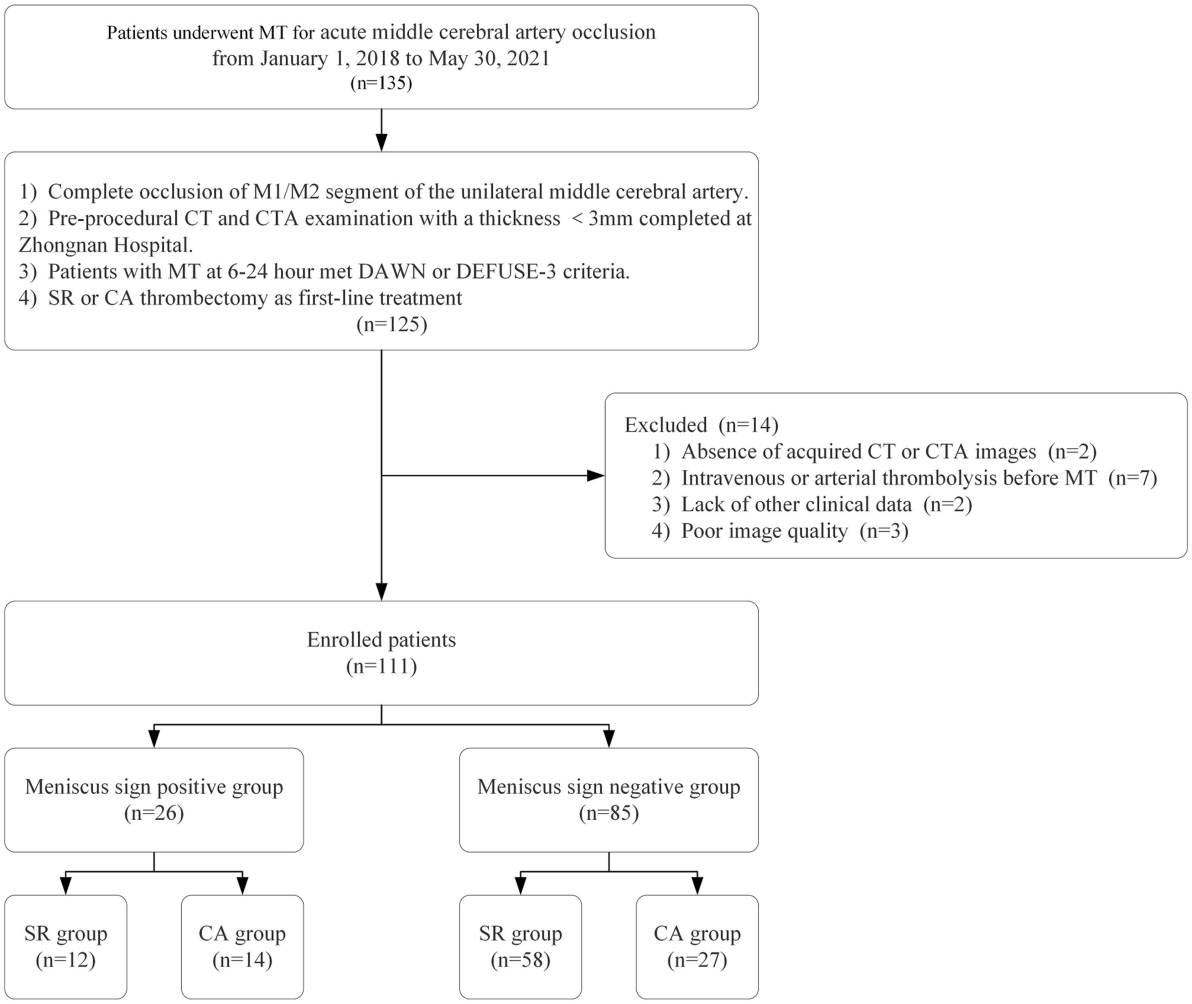
Flow diagram of the study. MT, mechanical thrombectomy; SR, stent retriever; CA, contact aspiration; CTA, CT angiography.

We retrospectively collected data of patients with acute middle cerebral artery occlusion (MCAO) who underwent MT at Zhongnan Hospital of Wuhan University from January 1, 2018 to May 30, 2021. The inclusion criteria were the following: 1) complete occlusion of M1/M2 segment of the unilateral middle cerebral artery, 2) pre-procedural computed tomography (CT) and computed tomographic angiography (CTA) examination with a thickness <3 mm completed at Zhongnan Hospital, 3) patients with MT at 6-24 h met DAWN (2) or DEFUSE-3 (3) criteria, and 4) SR or CA thrombectomy as first-line treatment. Exclusion criteria were the following: 1) absence of acquired CT or CTA images, 2) intravenous or arterial thrombolysis before MT, 3) lack of other clinical data, and 4) poor image quality. Finally, 111 patients were enrolled in our study and divided into two groups based on the presence or absence of meniscus sign (Figure 1).

We collected the demographics and radiographic, clinical, and surgical data of the patients. The modified Thrombolysis in Cerebral Infarction (mTICI) grading scale (10) was used to assess revascularization after MT, and mTICI = 3 was defined as complete recanalization. Functional outcomes were evaluated by National Institutes of Health Stroke Scale (NIHSS) score at discharge and modified Rankin Scale (mRS) scores at 90 days (0–2 = good outcome, ≥3 = bad outcome). We determined the subtype of stroke by TOAST classification criteria (11) and adopted 12-channel electrocardiogram, 24-h electrocardiogram monitoring, echocardiography, carotid ultrasonography, and MRI scans. Ethics approval of this study was obtained from the Medical Ethics Committee of Zhongnan Hospital of Wuhan University.

### Meniscus Sign Definition

**Figure 2:**
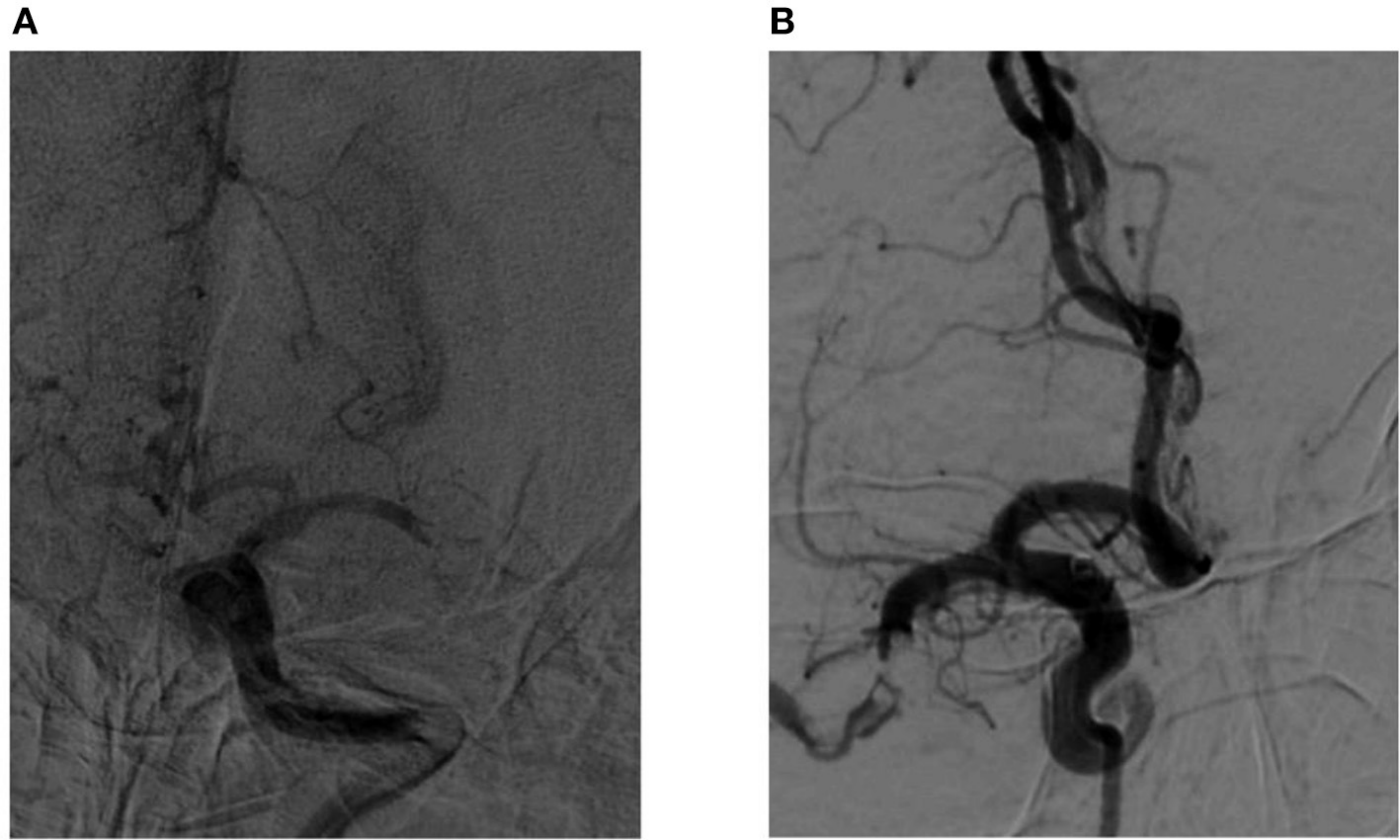
**(A,B)** Images of a middle cerebral artery occlusion in patients with positive meniscus sign.

Occlusive clot sign was defined as the angiographic appearance of the occluded site before recanalization, and the meniscus sign is a special type. Clot meniscus sign was defined as an appearance of meniscoid/edge-like or single- or double-wall contrast channels besides or around insular blood clots at the proximal end of the occluded vessel in DSA (7, 8) (Figure 2).

### Thrombus Permeability Assessment

**Figure 3:**
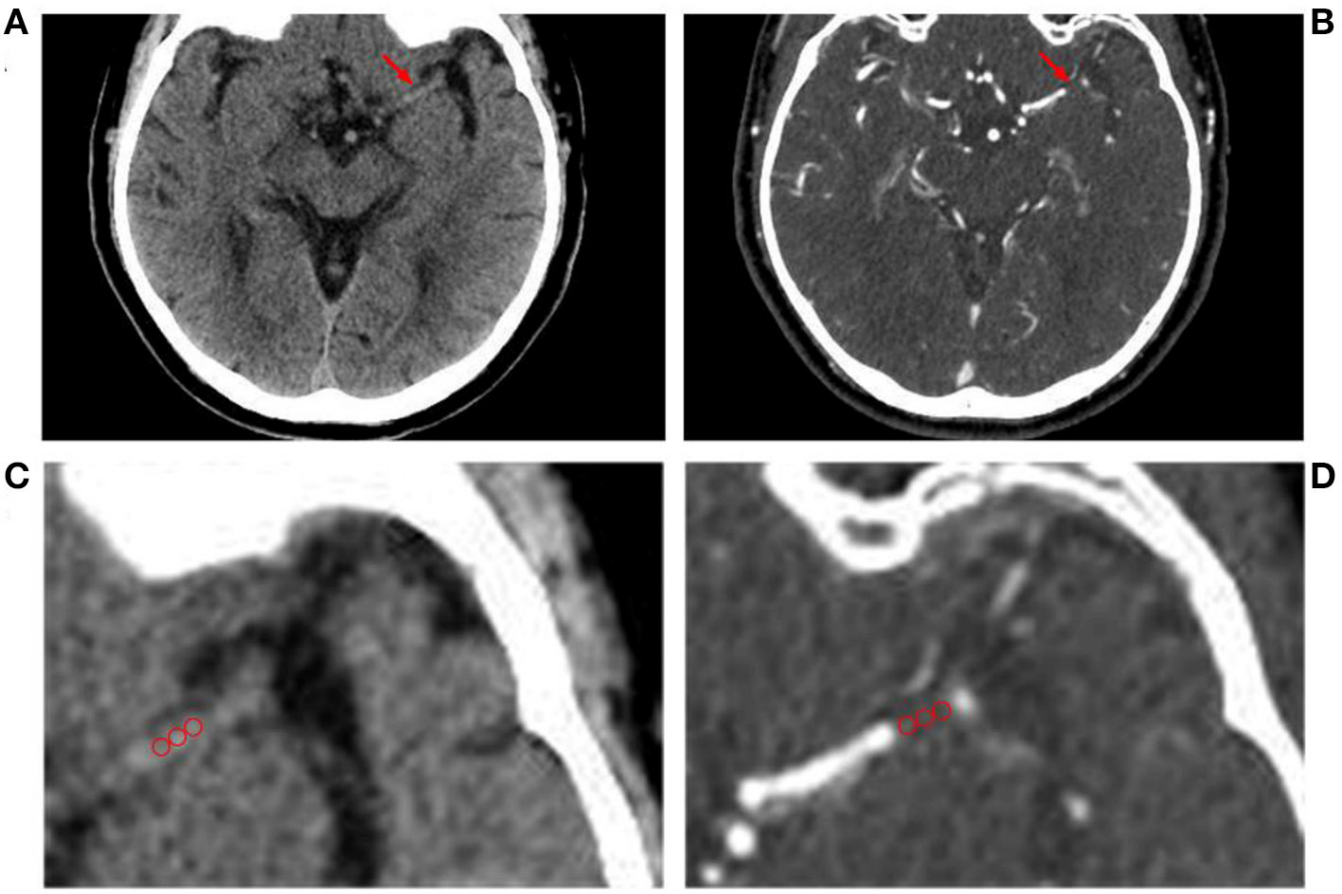
Measurement of thrombus permeability on non-contrast CT (nCT, A&C) and CT angiography (CTA, B&D). Three regions of interest (red circles) are placed in the proximal, middle, and distal parts of the thrombus (red arrow) to calculate the mean Hounsfield unit (HU) density value of the occlusion site. **(C,D)** Are partial magnifications of **(A,B)**.

Thrombus permeability was assessed by the uptake of contrast medium on arterial CTA and difference in Hounsfield units (HUs) between non-contrast CT (nCT) and CTA (5, 12). The measurement steps were as follows: (1) alignment of nCT images with CTA scans can be automated by the system and evaluated by a neuroradiologist with at least 2 years of experience (MT). (2) In consideration of growth of the appositional thrombus, the point of measurement was selected to be 1.5 mm behind the occluded site, and three circular selection circles with a 1-mm radius were used to measure the occlusion site. (3) Mean HU difference between CTA and nCT was defined as thrombus perviousness Δt = HU_CTA_− HU_nCT_ (Figure 3).

### Endovascular Treatment

All the patients underwent MT and were divided into different groups according to the first treatment with SR or CA. All endovascular treatments were completed by 2 interventional neuroradiologists with more than 5 years of interventional experience (BM. and DS). The first-line thrombectomy method was selected by the interventional neuroradiologists during the operation. The retrievers used in the study included Solitaire FR (Medtronic, Minneapolis, MN, United States), Solitaire AB (Medtronic, Minneapolis, MN, United States), and Trevo (Stryker Neurovascular, Salt Lake City, UT, United States). A large-bore aspiration catheter (Penumbra System Reperfusion Catheter ACE™ 60; Penumbra, CA, United States) or an intermediate catheter was used in CA. If the first-line method initially selected failed to achieve effective recanalization, another method would be used for rescue treatment. All the patients had completed CT and CTA examinations and signed informed consent before surgery.

### Statistical Analysis

In all samples, the quantitative variable was expressed as mean ± standard deviation or median with interquartile range, and the qualitative variable was expressed as counts and frequencies. Pearson χ^2^ tests or Fisher exact tests were performed for categorical variables, and continuous variables were compared by *t*-tests or the Mann–Whitney *U* test. Multivariate logistic regression was used to evaluate the independent variables of complete recanalization in patients with positive meniscus sign. *P* < 0.05 was considered statistically significant. Statistical analyses were performed using SPSS for Windows (Version 23.0; IBM).

## Results

**Figure 4:**
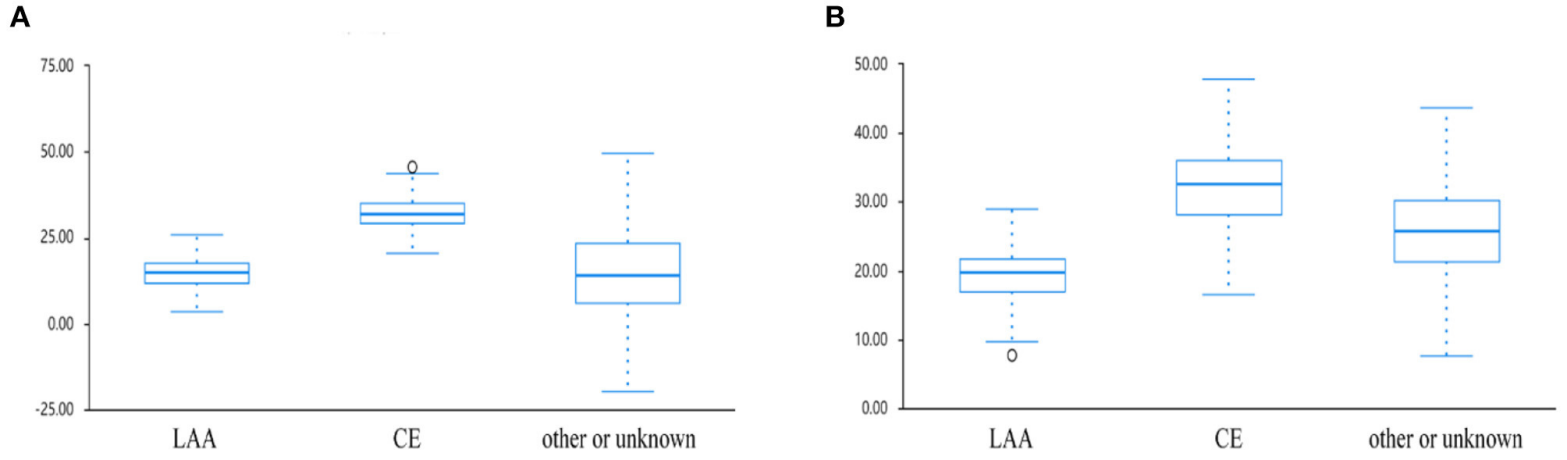
Boxplots of change in thrombus permeability for different etiological groups in patients **(A)** with or **(B)** without meniscus sign.

**Figure 5:**
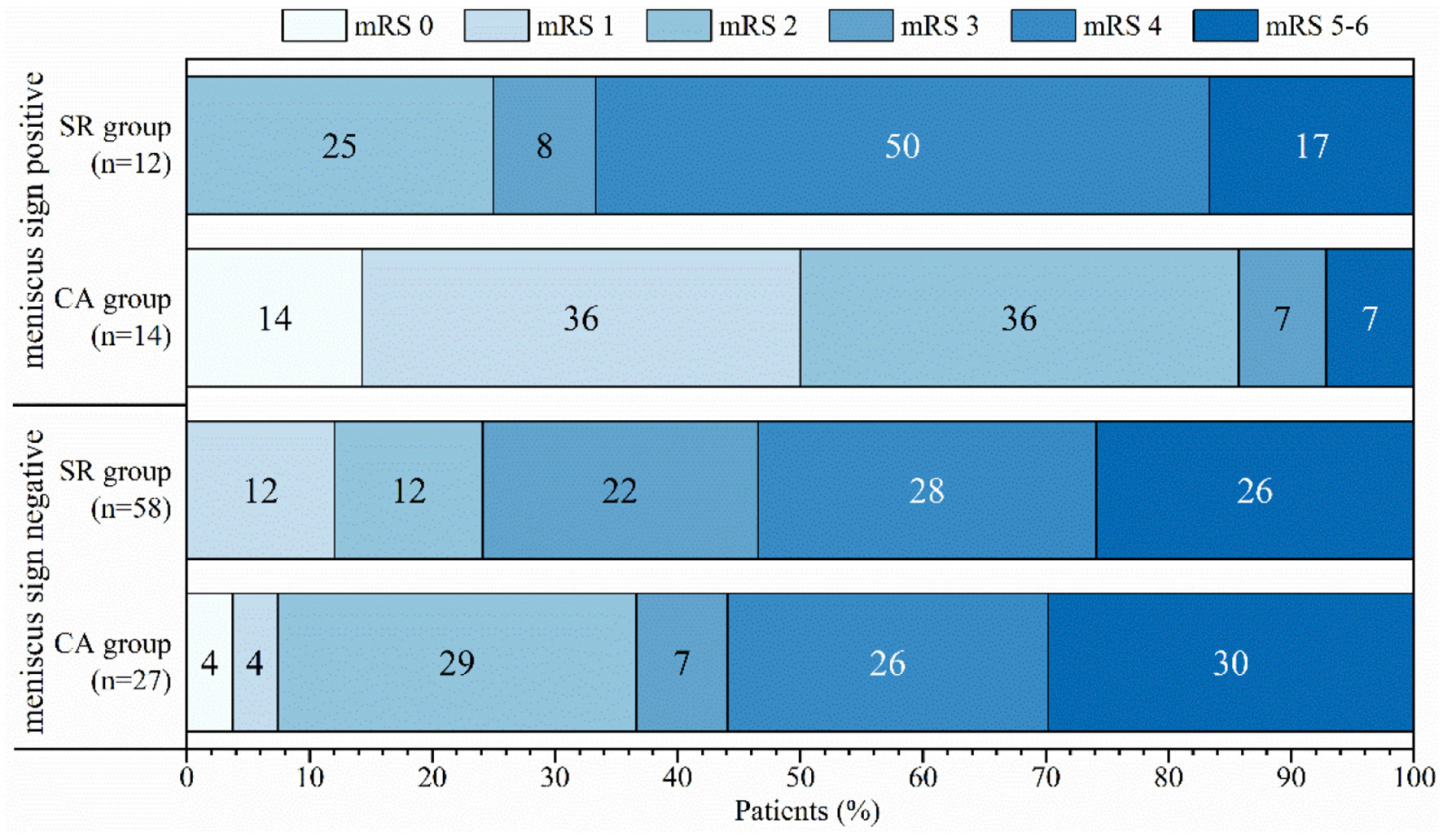
Distribution of mRS score at 90 days among different groups.

A total of 111 patients who met our inclusion criteria were examined in the study, and included 54 males and 57 females. Compared with the negative group, the meniscus sign positive group had higher prevalence of atrial fibrillation (AF) (21.18 vs. 46.15%, *p* = 0.012), cardiogenic embolism (CE) (23.53 vs. 69.23%, *p* < 0.01), and use of CA (31.76 vs. 53.85%, *p* = 0.041). The group with meniscus sign had higher thrombus permeability (HUs) (22.84 ± 7.88 vs. 26.92 ± 9.69, *p* = 0.031) and better outcome (28.24 vs. 57.69%, *p* < 0.01) than the group without meniscus sign (Table 1). The thrombus permeability of different stroke subtypes in the meniscus sign positive and negative groups is shown in Figure 4. In patients with meniscus sign (*n* = 26), the CA group (*n* = 14) had shorter puncture-to-recanalization (P2R) time (88 vs. 65.5, *p* = 0.012), higher frequency of complete recanalization (33.33 vs. 85.71%, *p* < 0.01), higher rate of mRS score of 0–2 at 90 days (25 vs. 85.71%, *p* < 0.01), and lower NIHSS score at discharge (11.5 vs. 6, *p* = 0.037) (Table 2 and Figure 5). However, there was no statistically significant result in different MT groups in patients without meniscus sign (Table 3 and Figure 5). By multivariate regression analysis, CA was significantly associated with complete recanalization in patients with meniscus sign, with an odds ratio of 16.85 (95% confidence interval 1.409–201.486; *p* = 0.026) (Table 4).

**Table 1:** Comparison of data between patients with and without meniscus sign.

	**Meniscus sign** **negative** **(***n*** = 85)**	**Meniscus sign** **positive** **(***n*** = 26)**	***P*** **-values**
Age, years	65.25 ± 13.20	67.96 ± 13.34	0.362
Sex, male, *n* (%)	44 (51.76)	10 (38.46)	0.235
Medical history			
Hypertension, *n* (%)	44 (51.76)	16 (61.54)	0.382
Diabetes mellitus, *n* (%)	14 (16.47)	5 (19.23)	0.744
Dyslipidemia, *n* (%)	8 (9.41)	0 (0.00)	0.104
Atrial fibrillation, *n* (%)	18 (21.18)	12 (46.15)	0.012*
Coronary disease, *n* (%)	17 (20.00)	4 (15.38)	0.599
Smoking, *n* (%)	18 (21.18)	9 (34.62)	0.162
Past ischemic stroke, *n* (%)	14 (16.47)	3 (11.54)	0.541
Stroke subtype			
LAA, *n* (%)	58 (68.24)	5 (19.23)	<0.01*
CE, *n* (%)	20 (23.53)	18 (69.23)	<0.01*
Other or unknown, *n* (%)	7 (8.24)	3 (11.54)	0.607
Baseline mRS score, median (IQR)	0 (0, 0)	0 (0, 0)	0.433
Baseline NIHSS score, median (IQR)	17 (13.0, 21.5)	17.5 (11.8, 20.5)	0.810
Thrombus permeability (Hus)	22.84 ± 7.88	26.92 ± 9.69	0.031*
Surgical data			
O2Ptime, min, median (IQR)	455.0 (321.5, 609.0)	472.5 (307.5, 598.5)	0.892
P2R time, min, median (IQR)	89.0 (66.5, 117.5)	76.5 (46.3, 93.0)	0.056
MT concepts, CA, *n* (%)	27 (31.76)	14 (53.85)	0.041*
Complete recanalization, mTICI = 3, *n* (%)	47 (55.29)	16 (61.54)	0.574
Clinical outcomes			
mRS score of 0–2 at 90 days, *n* (%)	24 (28.24)	15 (57.69)	<0.01*
NIHSS score at discharge, median (IQR)	11 (6.0, 26.0)	8 (3.8, 14.3)	0.092

* *Variables with p value < 0.05*.

*LAA, large artery atherosclerosis; CE, cardiogenic embolism; NIHSS, National Institutes of Health Stroke Scale; mRS, modified Rankin Scale; O2P, onset-to- puncture; P2R, puncture-to-recanalization; MT, mechanical thrombectomy; CA, contact aspiration; mTICI, modified Thrombolysis in Cerebral Infarction*.

**Table 2:** Comparison of surgical data and clinical outcome among patients with meniscus sign in different MT methods.

	**Meniscus sign** **positive** **(*****n*** **= 26)**		***P*** **-values**
	**SR (***n*** = 12)**	**CA (***n*** = 14)**	
O2P time, min, median (IQR)	485.0 (251.3, 617.3)	460.0 (381.3, 599.8)	0.719
P2R time, min, median (IQR)	88.0 (84.3, 104.3)	65.5 (40.5, 75.3)	0.012*
Complete recanalization, mTICI = 3, *n* (%)	4 (33.33)	12 (85.71)	<0.01*
mRS score of 0–2 at 90 days, *n* (%)	3 (25.00)	12 (85.71)	<0.01*
NIHSS score at discharge, median (IQR)	11.5 (5.8, 20.5)	6.0 (3.0, 9.8)	0.037*

* *Variables with p value <0.05*.

*SR, stent retriever; CA, contact aspiration*.

**Table 3:** Comparison of surgical data and clinical outcome among patients without meniscus sign in different MT methods.

	**Meniscus sign** **negative** **(*****n*** **= 85)**		***P*** **-values**
	**SR (***n*** = 58)**	**CA (***n*** = 27)**	
O2P time, min, median (IQR)	470.5 (352.5, 646.5)	435.0 (232.0, 560.0)	0.216
P2R time, min, median (IQR)	95.5 (70.0, 121.3)	74.0 (52.0, 112.0)	0.308
Complete recanalization, mTICI = 3, *n* (%)	28 (48.28)	19 (70.37)	0.056
mRS score of 0–2 at 90 days, *n* (%)	14 (24.14)	10 (37.04)	0.219
NIHSS score at discharge, median (IQR)	11.0 (6.0, 25.0)	11.0 (5.0, 35.0)	0.868

*SR, stent retriever; CA, contact aspiration*.

**Table 4:** Multivariate regression analysis for complete recanalization in patients with meniscus sign.

	**OR**	**95% CI**	***P*** **-values**
O2P time, min	1.003	0.996–1.010	0.351
P2R time, min	1.002	0.963–1.043	0.909
MT methods, CA, *n* (%)	16.850	1.409–201.486	0.026*
Thrombus permeability	1.078	0.932–1.247	0.314

* *Variables with p value < 0.05*.

## Discussion

This study showed that the presence of meniscus sign was associated with AF, CE, higher thrombus permeability, and better clinical outcome. In patients with positive meniscus sign, shorter P2R time, higher complete recanalization rate, and better prognosis could be obtained performing CA.

To clarify how to choose MT methods to obtain better function outcomes, we analyzed the relationship between the morphological characteristics of thrombus and various clinical data. According to the morphology of proximal thrombus occlusion, it can be divided into cutoff, claw, meniscoid, tram-track, or tapered (7, 8, 13, 14). The meniscus sign, another subtype of claw sign, is usually combined with the tram-track sign as irregular occlusive clot signs. Existing studies have shown that the imaging characteristics similar to the meniscus sign are more likely to have a history of AF and related to the embolization mechanism (14), which is consistent with our research results. A study on clinical data of 187 patients with occlusion of the carotid-T or middle cerebral artery who underwent MT has confirmed the composition characteristics of high fibrin and low RBC in cardiogenic thrombosis (15), which is consistent with the results of previous studies of Boeckh-Behrens et al. (16) and Simons et al. (17). The study of Berndt et al. (5) has shown that high thrombus permeability is strongly correlated with lower RBC count and more fibrin, which can predict CE. However, the relationship with thrombus occlusion morphology has not been further explored. Based on this, our research further analyzed the relationship between meniscus sign and thrombus permeability, and confirmed a significant correlation between the two, and this may be helpful in determining the subtype of stroke and making decisions on MT methods.

As the first prospective randomized controlled study comparing SR and CA, the ASTER study showed that there was no significant difference in the effect of CA on the prognosis of patients compared with SR (18). The study by Martini et al. (19) has confirmed that the time from puncture to recanalization was shorter in patients with acute anterior circulation stroke in the CA group than in the SR group. This result is consistent with those of studies such as COMPASS (20) and ASTER (18), but the preoperative mRS score of the CA group was higher.

Based on the morphological phenotype of the occlusion site, there are limited studies available on the efficacy of different MT methods. Consoli et al. (21) classified that patients with irregular occlusion had higher rate of adequate recanalization after receiving the SR treatment rather than CA in M1-middle cerebral artery occlusion. Baik et al. (22) reported that CA as a first-line MT method may be more effective than SR in terms of complete recanalization in patients with basilar artery occlusion and clot meniscus sign.

In our study, patients with meniscus sign who underwent CA have better recanalization efficacy and clinical outcome compared to those who received an SR, while there was no statistical significance in the meniscus sign negative group. There are several possible reasons for the superiority of CA over SR. First, the efficacy of different MT methods depends on the mechanism of embolus formation. Clots rich in RBC break down more easily than those rich in fibrin (23), which is considered to be related to the embolic mechanism and suitable for CA. The formation of meniscus sign may be related to a clot moving to the occlusion, causing the contrast agent to form a channel on single/double sides of the blood vessel wall (14). Second, the CA procedure is started by advancing a guide catheter that contains an 8- or 9-Fr balloon guiding catheter (BGC) or 6-Fr neurosheath to the proximity of the occluded target vessel, while meniscus-like appearance provides a larger area for contact with the catheter (24). Choosing CA with proximal blood flow arrest by BGC can reduce the risk of ectopic distal thrombosis and increase the effect of flow reversal during aspiration (25–27).

There are several limitations in this study. First, on account of the retrospective design, the choice of first-line MT is completely determined by the surgeon based on clinical experience. Second, this study lacks histopathological data on thrombus and ignores the influence of different types of contrast agents when measuring thrombus permeability. Finally, this study is a single-center one with small sample size, and a multi-center and large-sample prospective study should be carried out in the future.

## Conclusions

Among patients with MCAO, those with positive meniscus sign have higher thrombus permeability and are more suitable for CA as the first-line MT method to obtain higher complete recanalization rate and better functional outcomes.

## Data Availability Statement

The original contributions presented in the study are included in the article/supplementary material, further inquiries can be directed to the corresponding author.

## Ethics Statement

The studies involving human participants were reviewed and approved by the Medical Ethics Committee of Zhongnan Hospital of Wuhan University (approval number: 2020198). Written informed consent was not required for the current study in accordance with the local legislation and institutional requirements.

## Author Contributions

CN and BM designed the study. CN and ZK collected the data of the patients. CN analyzed the data and drafted the manuscript. BM, DS, XW, and MT performed the treatment procedure, interpreted the data, and revised the manuscript. All authors contributed to the article and approved the submitted version.

## Funding

This work was supported by the Translational Medicine and Interdisciplinary Research Joint Fund of Zhongnan Hospital of Wuhan University (No. ZNJC201924).

## Conflict of Interest

The authors declare that the research was conducted in the absence of any commercial or financial relationships that could be construed as a potential conflict of interest.

## Publisher's Note

All claims expressed in this article are solely those of the authors and do not necessarily represent those of their affiliated organizations, or those of the publisher, the editors and the reviewers. Any product that may be evaluated in this article, or claim that may be made by its manufacturer, is not guaranteed or endorsed by the publisher.
